# Incretin Hormones in Obesity and Related Cardiometabolic Disorders: The Clinical Perspective

**DOI:** 10.3390/nu13020351

**Published:** 2021-01-25

**Authors:** Joanna Michałowska, Ewa Miller-Kasprzak, Paweł Bogdański

**Affiliations:** Department of Obesity and Metabolic Disorders Treatment and Clinical Dietetics, Poznań University of Medical Sciences, Szamarzewskiego St. 82/84, 60-569 Poznań, Poland; emilka@ump.edu.pl (E.M.-K.); pbogdanski@ump.edu.pl (P.B.)

**Keywords:** obesity, GLP-1, GIP, incretins, incretin-based therapy, type 2 diabetes mellitus, non-alcoholic fatty liver disease, cardiovascular disease

## Abstract

The prevalence of obesity continues to grow rapidly worldwide, posing many public health challenges of the 21st century. Obese subjects are at major risk for serious diet-related noncommunicable diseases, including type 2 diabetes mellitus, cardiovascular disease, and non-alcoholic fatty liver disease. Understanding the mechanisms underlying obesity pathogenesis is needed for the development of effective treatment strategies. Dysregulation of incretin secretion and actions has been observed in obesity and related metabolic disorders; therefore, incretin-based therapies have been developed to provide new therapeutic options. Incretin mimetics present glucose-lowering properties, together with a reduction of appetite and food intake, resulting in weight loss. In this review, we describe the physiology of two known incretins—glucose-dependent insulinotropic polypeptide (GIP) and glucagon-like peptide-1 (GLP-1), and their role in obesity and related cardiometabolic disorders. We also focus on the available and incoming incretin-based medications that can be used in the treatment of the above-mentioned conditions.

## 1. Introduction

The prevalence of obesity is rapidly growing worldwide, posing serious consequences to individuals, society and the economy [1,2]. This disease is associated with many comorbidities, being one of the leading causes of mortality and morbidity. Obese adults are at higher risk of developing type 2 diabetes mellitus (T2DM), cardiovascular disease (CVD), non-alcoholic fatty liver disease (NAFLD) and other health problems [3,4,5]. Therefore, understanding the key mechanisms involved in the pathogenesis of obesity is needed for the development of prevention and therapeutic strategies. Research evidence shows that gastrointestinal (GI) tract hormones incretins may play a vital role in the pathogenesis and treatment of obesity and its comorbidities, as they are responsible for the regulation of body weight, maintenance of energy balance, and glucose homeostasis [6,7,8,9].

**Figure 1:**
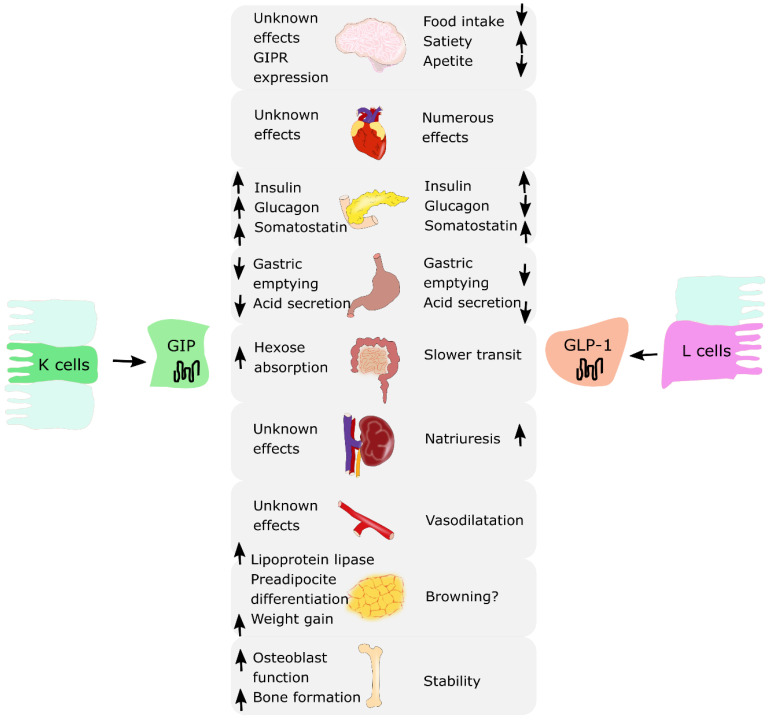
The summary of biological effects of glucose-dependent insulinotropic polypeptide (GIP) and glucagon-like peptide-1 (GLP-1) on various organs. ↑: increase; ↓: decrease.

Incretin hormones are released from the intestine after nutrient intake. They play a crucial role in stimulating insulin and glucagon secretion by the pancreas [10,11]. There are two known incretins: glucose-dependent insulinotropic polypeptide (GIP) produced by the K cells of an upper gut and glucagon-like peptide-1 (GLP-1) produced by the L cells of a lower gut. Together, they are responsible for an “incretin effect”, which refers to the observation of two- to three-fold higher insulin secretion after oral glucose intake, in comparison to an equivalent intravenous glucose administration [8,9]. Fasting healthy subjects have low basal plasma concentrations of GIP and GLP-1. They start to rise few minutes after nutrient ingestion, reaching a peak approximately after 1 h, with GIP concentrations being usually higher than GLP-1 concentrations. In patients with T2DM, the incretin effect is diminished or absent. This is due to the fact that the pancreas is no longer responsive to GIP; however, it does remain sensitive to GLP-1 [12]. Thus, incretin-based glucose-lowering medications, in particular GLP-1 receptor agonists (GLP-1RAs), have proven to be effective and are currently used in T2DM treatment [13,14,15,16,17,18]. A similar observation of decreased incretin effect was made in obese individuals with normal glucose tolerance. Several studies reported a reduction in the incretin effect in nondiabetic individuals with obesity, suggesting that incretin hormones may play a role in the pathophysiology of obesity [9,19,20]. Moreover, incretins present pleiotropic actions and effects in various organ systems. GLP-1 is responsible for the reduction in food intake and appetite, increased satiety, and decreased gastric emptying. It affects adipose cells, bone metabolism and the cardiovascular system. GIP addresses fewer organs and functions compared to GLP-1; however, research suggests that it may also influence adipose tissue by promoting fat storage in subcutaneous adipose tissue and bone metabolism by promoting bone formation and limiting bone resorption [8,9]. The summary of the biological effects of GIP and GLP-1 on various organs is presented in Figure 1.

Incretin hormones play an important role in human physiology, presenting different actions by peripheral and central mechanisms. The aim of this review is to describe and summarize the current knowledge regarding the role of incretin hormones in the pathophysiology of obesity and associated cardiometabolic disorders; and their therapeutic potential.

## 2. Biology of Incretins

The term incretin was introduced in 1932 to describe compounds produced by intestinal mucosa in response to nutrient ingestion, which were capable of reducing blood glucose [21]. GIP was discovered in 1970 by John C. Brown in dogs. This hormone was initially named “gastric inhibitory polypeptide”, due to its inhibitory effect on gastric acid secretion [22,23]. The insulinotropic action of GIP in humans was presented three years later, and the acronym GIP was proposed to be changed to its current name—glucose-dependent insulinotropic polypeptide [24,25]. The work in the next several years was focusing on the role of GIP in the pathogenesis of T2DM and its potential for the treatment of this disease, and the evidence that this peptide is not the only incretin emerged [25]. Finally, in 1980s glucagon-like peptides (called GLP-1 and GLP-2) were identified by cloning of the preproglucagon gene, which exhibited insulin-releasing activity. Further investigation demonstrated that truncated GLP-1 (7–36) shows not only insulinotropic effect but also inhibits the secretion of glucagon [21,25].

GIP is a peptide, which is synthesized and secreted mainly from K cells located in the duodenum and proximal jejunum; however, expression of this incretin by the central nervous system (CNS) has also been observed [26]. GIP expression has been detected in the brain, including hippocampus, thalamus, cerebellum, brainstem, and cortex in rats [27], and hypothalamus in humans [28]. This incretin derives from a precursor pro-peptide (pro-GIP), which is posttranslationally processed at residue 65 by proprotein convertase subtilisin/kexin type 1 to 42 amino acid (aa) form of GIP(1–42). Another amidated isoform of GIP(1–30) has also been detected as the pro-GIP peptide sequence contains a consensus cleavage site for prohormone convertase 2 (PC2) at residues 52–55 [29]. Both isoforms bind to GIP receptor (GIPR) and present intrinsic activity; however, it was speculated that they might have different pharmacokinetic profiles [30].

**Figure 2:**
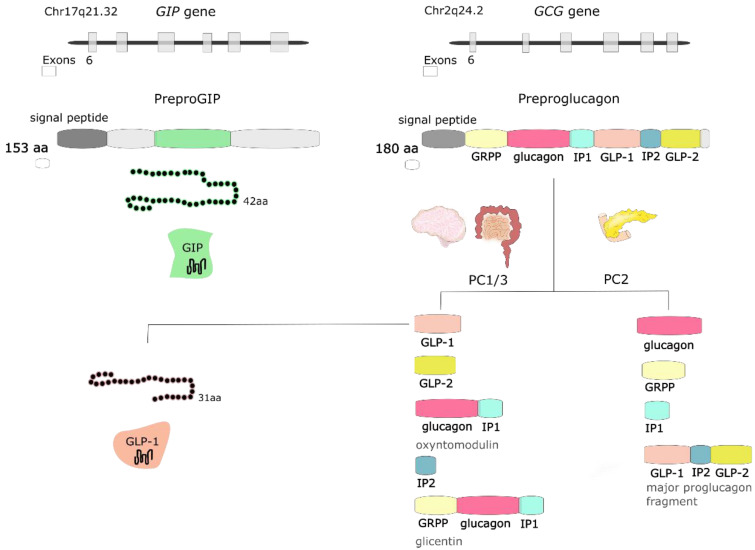
The *GIP* gene is localized on human chromosome Chr17q21.32 and encodes 153-amino acid (aa) proprotein, which is processed to 42-aa protein glucose-dependent insulinotropic peptide (GIP). *GCG* gene is localized on human chromosome Chr2q24.2 and encodes preproglucagon—180-aa protein, which undergoes differential posttranslational processing in a particular type of cell. Proteolytic processing of proglucagon by prohormone convertase 1/3 (PC1/3) in the intestine generates glucagon-like peptide 1 (GLP-1), glucagon-like peptide 2 (GLP-2), oxyntomodulin, intervening peptide 2 (IP2) and glicentin. Prohormone convertase 2 (PC2) activity in the pancreas forms glucagon, glicentin-related pancreatic polypeptide (GRPP), intervening peptide 1 (IP1), and major proglucagon fragment.

GLP-1 is synthesized and secreted mainly from L cells located in the small and large intestine, but it is also expressed in the CNS, primarily in the brainstem [26]. *GCG* gene encodes preproglucagon, which is cleaved to form proglucagon—160-aa long protein that undergoes differential posttranslational processing in particular types of cells, based on the relative activities of the prohormone convertases 1/3 (PC1,3) and 2. In L-cells of the intestine and specific CNS neurons, PC1 predominates, and proglucagon is cleaved to five functional regions: two carboxy-terminal glucagon-like peptides (GLP-1 and GLP-2), oxyntomodulin, glicentin, and intervening peptide 2. In α cells of the pancreas, the action of PC2 is greater, and proglucagon is processed to glucagon, glicentin-related pancreatic polypeptide (GRPP), intervening peptide 1, and major proglucagon fragment [31] (Figure 2). There are two biologically active forms of GLP-1 classified as amidated GLP-1 (GLP-1(7–36) amide) and glycine-extended GLP-1 (GLP-1(7–37)) [8].

**Figure 3:**
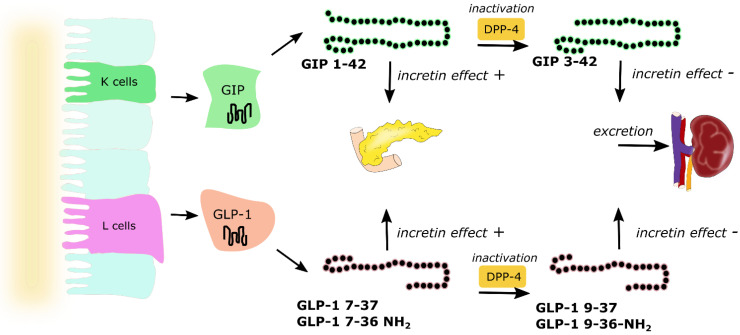
Secretion and metabolism of glucose-dependent insulinotropic peptide (GIP) and glucagon-like peptide 1 (GLP-1). GIP is secreted by K cells of the upper intestine, whereas GLP-1 is secreted by L cells of the lower intestine. Active forms of both incretins GIP(1–42), GLP-1(7–37) and amidated GLP-1(7–36) act directly on the pancreas and are responsible for the incretin effect. Both GIP and GLP-1 are inactivated by dipeptidyl peptidase 4 (DPP-4) to GIP(3–42), GLP-1(9–37) and GLP-1(9–36), which are excreted by the kidneys. Arrows represent pathways of incretins metabolism.

Glucose is one of the nutrients stimulating the secretion of GIP and GLP-1; however other nutrients, including carbohydrates (sucrose, starch), triglycerides, some amino acids and proteins, are also responsible for increasing incretin levels [32]. About two-thirds of the insulin response to an oral glucose load is a result of incretin hormone release, where both GIP and GLP-1 stimulate insulin secretion in a glucose-dependent manner [33]. They exert their effect by binding to their specific receptors—GIPR and GLP-1R, which belong to the G-protein coupled receptor family [34]. GIPR and GLP-1R stimulation activates adenylate cyclase and results in the increase of intracellular cyclic adenosine monophosphate (cAMP), leading to the activation of protein kinase A (PKA) and cAMP-regulated guanine nucleotide exchange factor II (cAMP-GEFII). These two proteins regulate insulin release via the formation of ATP, closure of ATP-sensitive K+ (KATP) channels, β-cell depolarization, the opening of voltage-dependent Ca^2+^ channels, the influx of the ions and elevation of the intracellular Ca^2+^ concentration, which triggers exocytosis of insulin granules. This mechanism accounts for approx. 70% of total insulinotropic activity exerted by both incretins [33]. Except for insulinotropic activity, incretin hormones affect pancreatic α cells and glucagon secretion. The evidence indicates that GIP stimulates glucagon release, whereas GLP-1 inhibits glucagon secretion (especially at high glucose concentrations). The glucagonostatic effect of GLP-1 leads to reduced hepatic glucose production [8]. Both GIP and GLP-1 are inactivated by dipeptidyl peptidase-4 (DPP-4), which converts active forms to inactive GIP(3–42), GLP-1(9–36) amide and/or GLP-1(9–37), which are subsequently excreted from the kidneys (Figure 3). DPP-4 processed incretins do not exhibit insulinotropic effect; however, recent studies showed that those shorter forms might have some additional biologic activities, as truncated forms of GLP-1—GLP-1(9–36) amide and GLP-1(9–37) have been demonstrated to be associated with GLP-1 cardioprotective properties [34,35].

## 3. Incretin Hormones in Obesity

### 3.1. Biological Functions of Incretins Associated with Food Intake and Weight

Activation of GLP-1R and GIPR affects multiple tissues; therefore, incretin hormones are responsible not only for regulating glucose levels but also influence lipid metabolism, gastrointestinal tract, appetite and body weight [8,26]. In this section, we are describing additional biological effects of incretin hormones markedly important for obesity pathogenesis and treatment.

#### 3.1.1. Appetite, Satiety and Food Reward

Administrating GLP-1 or GLP-1 mimetics—GLP-1RA, which act as GLP-1, both into general circulation or the CNS, decreases appetite, increases satiety and reduce food intake [36,37,38,39,40,41]. This has been confirmed on animal models [42,43,44,45], as well as in various human populations, including lean individuals [46,47] and individuals with obesity [48,49,50,51]. However, not all of the studies confirmed the influence of incretin hormones on food intake. Ten Kulve et al. found that administration of GLP-1RA liraglutide decreases CNS activation only during short-term treatment in obese individuals with T2DM. After 10 days, patients treated with liraglutide showed decreased responses to food pictures in insula and putamen in comparison to the control group treated with insulin glargine. The enhanced satiating effect of meal intake on responses in the putamen and amygdala was also observed. There were no differences between groups after 12 weeks of treatment; therefore, the authors suggested GLP-1RA’s role in the introduction of weight loss, but not weight loss maintenance [52]. On the other hand, administration of GLP-1RA in otherwise healthy individuals with obesity was not associated with increased satiety after 16 weeks of treatment [53]. Long et al. also showed no effect of centrally administrated GLP-1 on short-term satiety in non-obese men [54]. Future research should focus on individual differences in GLP-1 satiating effect to determine responsiveness to treatment and to establish which patients could benefit from this type of therapy.

Hypothalamic and brainstem response to GLP-1R activation seems to be the crucial factor responsible for GLP-1 action influencing food intake [55,56,57,58]. GLP-1 directly stimulates anorexigenic proopiomelanocortin/cocaine and amphetamine-regulated transcript (POMC/CART) neurons and indirectly inhibits orexigenic neuropeptide Y/Agouti-related peptide (NPY/AgRP) neurons. Therefore, GLP-1 inhibits appetite and increases satiety [11,59]. This effect is further enhanced, as GLP-1 also plays a role in hedonic control of food intake [60]. Both endogenous and exogenous GLP-1 affects reward-related brain regions, including the mesolimbic reward system, insular cortex, and putamen [61,62,63]. As shown by Bloemendaal et al., emotional eaters are less sensitive to the central effects of GLP-1R activation; therefore, they have altered brain responses to food-cues [64]. In the study including obese subjects with T2DM, and lean and obese subjects with normoglycemia, GLP-1 receptor activation was associated with a decreased anticipatory food reward, as an infusion of the GLP-1RA decreased the anticipation for receiving chocolate milk, compared to placebo [65].

A study by Edholm et al. demonstrated that GIP infusion does not affect appetite, hunger, satiety and food intake [37]. On the contrary, Daousi et al. reported that healthy subjects declared higher hunger scores after GIP infusion [66]. However, it has been suggested that hypothalamic GIPR may play a role in energy balance [28]. The combination of GLP-1 and GIP agonists for improved treatment of obesity has also been studied; however, the results are not consistent [67,68,69,70]. Further studies are needed to establish the influence of GLP-1 and GIP dual agonists on food intake, appetite and satiety.

#### 3.1.2. Adipose Tissue

GIP has been proposed as one of the links between overfeeding and obesity. Research showed that mice lacking *GIPR* are prevented from developing obesity induced by a high-fat diet (HFD) [71,72]. Moreover, results from animal studies suggest that GIP may promote fat storage in subcutaneous adipose tissue as it induces lipoprotein lipase (LPL), promotes the elimination of chylomicron triglycerides, and inhibits catecholamine- and glucagon-induced lipolysis [73]. However, the excess fat deposition has not been confirmed in humans, and it was observed in animals only when fed HFD. The influence of GIP on adipose tissue and fat metabolism has been recently described elsewhere [74]. It is still not clear if GIP has a beneficial or detrimental impact on fat storage. The current evidence suggests that GIP is responsible for the incorporation of non-esterified fatty acids into adipose tissue and probably influences fat deposits in other tissues, e.g., the liver. The role of GIP in inducing LPL may be related to insulin deficiency; however, this has not been confirmed in humans [74]. Further research is needed to verify the therapeutic potential of GIP antagonism in obesity treatment.

GLP-1 has been suggested to play a beneficial role in fat metabolism by targeting subcutaneous, visceral and epicardial adipose tissue. Both animal and human studies showed that GLP-1RA reduces white adipose tissue (WAT) thickness, enhances WAT browning and induces adipocyte hyperplasia [6]. Randomized controlled trials showed that treatment with GLP-1RA—liraglutide is associated with a decrease in visceral fat in obese patients with T2DM or prediabetes [75,76,77,78]. Moreover, some studies showed that GLP-1R signaling affects brown adipose tissue (BAT) and increases thermogenesis, therefore influencing energy expenditure [79,80].

#### 3.1.3. Energy Expenditure

Animal studies demonstrated that exogenous activation of brain GLP-1R signaling by pharmacological administration increases energy expenditure (EE) in mice. Lockie et al. showed that intracerebroventricular injection of GLP-1 increases interscapular BAT thermogenesis [81]. The same effect was demonstrated after peripheral administration of GLP-1RA exendin-4 in rats [82]. Consistent with these results, other studies in mice revealed that central injection of liraglutide stimulates BAT thermogenesis and adipocyte browning through hypothalamic AMP-activated protein kinase (AMPK) [80], and the direct administration of GLP-1 into the dorsomedial hypothalamus (DMH) increases BAT thermogenesis through DMH GLP-1R signaling [83]. Several studies showed that endogenous GLP-1R signaling contributes to the control of EE of animals fed HFD [84,85], while Lockie et al. showed that *GLP-1R* knockout mice exhibit a normal response to cold exposure, suggesting that endogenous GLP-1R signaling is not essential for appropriate thermogenic response after cold exposure [81]. The effect of GLP-1 and GLP-1R agonists on EE in humans has not been established so far. In a systematic review of twenty-four trials that included 443 participants, the authors concluded that GLP-1 has no short-term effect on resting energy expenditure (REE) and may decrease diet-induced thermogenesis (DIT). Ten of included studies found no short-term change in REE after GLP-1 administration, two out of three trials reported a decrease in DIT after GLP-1 administration, and ten trials reported the neutral influence of GLP-1RA on REE, DIT and physical-activity induced EE. Only one study showed an increase in REE after GLP-1RA administration, and this trial had the longest duration. Despite no short-term effect on REE, further studies are needed to verify these results in prolonged treatment [86].

The effect of GIP on EE has been studied less extensively. Reduction of GIP secretion in HFD fed mice was associated with a decrease in the mass of adipose tissue, reduction of body weight gain, as well as with an increase in EE [87]. Consistent with these results, Hansotia et al. showed that *GIPR* knockout mice had enhanced locomotor activity, increased EE, and were resistant to diet-induced obesity [88]. Further research performed by this research team demonstrated that, in mice, GIPR is expressed in BAT, and GIP directly increases interleukin-6 (IL-6) mRNA and IL-6 secretion in BAT cells; however, the loss of the GIPR signaling within BAT alone was not associated with decreased weight gain and resistance to obesity [89]. Daousi et al. reported decreased resting EE and higher subjective appetite sensation in normal-weight patients after GIP infusion [66]. On the contrary, Asmar et al. showed that GIP does not affect EE in healthy males [90], while results of the study on patients with T2DM indicated that GIP infusion on top of the treatment with metformin and a long-acting GLP-1R agonist did not affect EE [91]. Study groups in described studies were relatively small; therefore, further studies are needed to establish the effect of GIP on energy expenditure in humans.

#### 3.1.4. Gastric Emptying and Intestinal Transit

Research shows that both physiological and pharmacological concentrations of GLP-1 reduce the rate of entry of nutrients into the circulation by a reduction of gastric emptying, which is an important mechanism for the satiating effect of this gut hormone. In rats, inhibition of gastric emptying occurs after peripheral or central administration of GLP-1 [92,93,94,95,96], and vagal afferent nerves have been suggested to mediate the inhibitory action of GLP-1 on gastric motor function [92]. Human studies also confirmed slower gastric emptying after administration of GLP-1 or GLP-1RA, both in healthy [37,97,98] and obese individuals [49,51,53,99]. However, Nauck et al. showed that GLP-1–induced delay in gastric emptying is subject to rapid tachyphylaxis as an intravenous administration of GLP-1 in healthy subjects inhibited gastric emptying to a greater extent after the first meal than after the second meal [100]. Similar results were presented in a randomized, double-blind, placebo-controlled pilot trial investigating the effects of liraglutide on weight, satiation, and gastric functions in obesity. In the study, treatment with liraglutide was associated with inhibition of gastric emptying after 5 weeks of treatment; however, this effect diminished after 16 weeks of treatment. Yet, the differences between treatment and placebo groups remained significant at week 5 and 16 [53].

Results of early studies showed that GIP is responsible for the inhibition of gastric acid secretion; therefore, it was suggested that it might inhibit gastrointestinal motility [22,23]. Nevertheless, further studies neither confirmed that GIP inhibits the secretion of gastric acid [101,102] nor that GIP has an influence on gastric emptying in humans [37,103,104].

### 3.2. Secretion and Action of GIP and GLP-1 in Obese, Nondiabetic Individuals

There is a growing body of evidence suggesting that increased body weight is associated with impairments in secretion and action of incretin hormones, as body mass index (BMI) has been identified as an independent factor associated with reduced GLP-1 secretion and an impaired incretin effect [19,105]. Findings of Knop et al. showed that the incretin effect was extensively more reduced in obese than in lean T2 diabetic individuals [20]. Fasting concentration of GLP-1 does not differ in lean and obese individuals [106], while secretion of this gut hormone is reduced in response to an oral glucose challenge. A number of studies have presented this phenomenon for postprandial GLP-1 responses; however, the results are not consistent [107,108]. The current evidence suggests that leptin resistance, impaired ghrelin secretion and hyperinsulinemia that occur in obesity cause functional deficits in GLP-1 signaling [107].

On the contrary, fasting and post-oral glucose tolerance test (OGTT) and GIP concentrations are higher in individuals with obesity than in lean individuals. This association has been observed, both in human [106,109] and animal studies [110]. In one of the studies, leptin-deficient obese *ob/ob* mice fed HFD showed increased concentration of GIP in plasma and intestine, whereas the density of GIP-secreting K cells in the upper jejunum increased by 54% as compared to the stock diet [111]. This may be due to increased stimuli from the gut lumen, such as an increase in gut endocannabinoid tone [10]. Hypersecretion of GIP in obesity may work as a compensatory mechanism; however, despite higher concentration, GIP cannot account for the hyperinsulinemia of obesity through its insulinotropic action [112].

### 3.3. Bariatric Surgery

Bariatric surgery is one of the methods of obesity treatment [113]. For patients with severe obesity, this procedure used in combination with behavioral interventions is the most effective in terms of long-term weight loss and control of comorbidities, including T2DM, hypertension and dyslipidemia [5,114]. Bariatric surgery results in major changes in the secretion and concentration of both incretin hormones, GLP-1 and GIP, as well as other gut hormones that are produced in the lower small intestine [8]. The concentration of GLP-1 rises rapidly and continues to be increased in patients after Roux-en-Y gastric bypass (RYGB), improving postprandial insulin secretion and glycemic control, which can be observed due to the faster delivery of nutrients to the lower intestine. The incretin effect in patients with T2DM is restored for years after the surgery and was shown to be weight loss independent, as the incretin effect is not restored in weight-matched subjects who lost 10% of weight by calorie restriction. The effect of RYGB on GIP concentration is less consistent, as this hormone is produced in the upper GI tract—the part that is bypassed due to the surgery. The differences in surgical techniques may therefore explain the differences in GIP concentrations in various studies [115]. A similar effect can be observed for patients undergoing sleeve gastrectomy. In this case, part of the stomach is removed, which leads to the rapid emptying of this organ and rapid nutrient delivery to the small intestine. In the randomized trial comparing the single anastomosis (mini-) gastric bypass (SAGB) and sleeve gastrectomy (SG), both groups had a similar percentage of weight loss, and an increase of the incretin effect was observed. However, this improvement was more pronounced in the first group [116]. Changes in the concentration of GIP and GLP-1 were not observed after adjustable gastric banding [117]. Studies where GLP-1R antagonist—exendin (9–39) was used in patients after RYGB showed that blocking GLP-1R was associated only with the limited deterioration of glucose tolerance [118,119,120]. Moreover, in *GLP-1R* knockout mice, body weight and glucose homeostasis improved after RYGB [121]. This suggests that the incretin effect and increased GLP-1 concentration after bariatric surgery may not be exclusive mediators of improved glycemic control, insulin sensitivity and weight loss that are achieved through this method of obesity treatment.

There was a hypothesis that an increase in GLP-1 concentration might be associated with the hypoglycemia that occurs as an uncommon complication in gastric bypass patients. The islet cell expansion due to the increased levels of GLP-1 was proposed as a suggested mechanism. This assumption was later rejected as animal studies did not show an increase in islet cellularity when using GLP-1RA doses >400 times higher than those used in humans [122]. Moreover, it has been suggested that the combination of GLP-1 analogs and bariatric surgery may result in better health outcomes. A recent systematic review looked into postoperative administration of GLP-1 analogs where authors concluded that the use of GLP-1 analogs in patients after bariatric surgery with unsatisfactory results might contribute to additional weight loss and improvements of comorbidities [123].

## 4. Incretin Hormones in Obesity-Related Metabolic Diseases

### 4.1. The Role of Incretins in T2DM

T2DM is one of the most common metabolic disorders. This disease is associated with insulin resistance and defective insulin secretion by the pancreas, leading to disruptions in glucose homeostasis [124]. As incretins are responsible for insulin secretion and play an important role in the regulation of glucose homeostasis, there has been a considerable interest to establish the role of GIP and GLP-1 in the pathophysiology of T2DM.

#### 4.1.1. Secretion of GLP-1 and GIP in Diabetic Individuals

Previous studies have shown that incretin levels and actions may be altered in T2DM; however, the results are unclear. The concentrations of GLP-1 have been reported to be elevated, decreased or unchanged, depending on the state of glucose control, duration of the disease and BMI status [9,10]. Two of the systematic reviews with meta-analyses concluded that in individuals with T2DM GLP-1 secretion in response to OGTT or a meal test is not reduced [125,126], where Calanna et al. concluded that poor glycemic control might be associated with reduced GLP-1 secretion [126]. Similar to GLP-1, GIP level also has been reported to be decreased, increased or unchanged in diabetic subjects [9]. A meta-analysis of 23 trials investigating GIP secretion after OGTT or mixed-meal test in T2DM patients showed that GIP secretion is preserved in diabetic patients with some exceptions—increased BMI was associated with increased GIP secretion, and higher age and glycated hemoglobin (HbA1c) were associated with reduced GIP level [127].

#### 4.1.2. Reduction in the Incretin Effect—Role of GIP and GLP-1 in the Pathophysiology of T2DM

There is a growing body of evidence suggesting that incretin concentrations may be predictors of prediabetes and T2DM development. Initial studies indicated that changes in secretion and action of incretins might play a role in the pathogenesis of T2DM; however, Meier et al. concluded that this association could not be confirmed [128]. The subsequent review investigated whether GLP-1 concentration may be a predictor of prediabetes. A total of 19 studies were included in the review, and the authors concluded that due to various results, it is not possible to establish if GLP-1 secretion and action is a predictor of T2DM. The authors highlighted the importance of carrying out prospective longitudinal studies to answer their research question [129]. Shortly after the publication, the first prospective observational study was published, in which authors examined the association between incretin responses to OGTT and mixed-meal test at baseline and changes in fasting glucose levels seven years later in individuals who were initially nondiabetic. The study included 121 subjects from the Hoorn Meal Study. The results showed no associations between GIP and changes in fasting glucose levels nor with a change in fasting glucose levels during follow-up. On the contrary, low GLP-1 response to OGTT was inversely associated with an increase in fasting glucose levels during seven years of follow-up. No significant associations were observed for GLP-1 responses following a mixed-meal test. Those results suggest that reduced GLP-1 secretion may actually play a role in the etiology of T2DM [130]. Further studies are needed to establish the causal role of GIP and GLP-1 secretion in the pathophysiology of T2DM.

Despite the fact that the concentration of GIP and GLP-1 in diabetic subjects seems to be preserved, the loss of incretin effect in those individuals is observed. It has been established that pancreatic islets in subjects with T2DM are no longer responsive to GIP and are only partially responsive to GLP-1. As GIP is responsible for a major part of the incretin effect, it is largely diminished or no longer present in patients with T2DM (despite the partially preserved GLP-1 function) [8,9,10,12]. Insulin response to physiological GLP-1 in diabetic patients is reduced compared with healthy individuals, most likely due to reduced functional β-cell mass [12]. Administration of exogenous GLP-1 is associated with three- to five-fold lower insulin response in diabetic subjects; however, GLP-1 administration in pharmacological doses is able to increase insulin secretion to normal levels and lower plasma glucose level [131]. Moreover, the glucagonostatic effect of GLP-1 is also preserved [132]. Those retained GLP-1 effects have a vast implication in T2DM treatment. Administration of exogenous GIP, even at high pharmacological doses, is not associated with a glucose-lowering effect in diabetic subjects [133]. Therefore, GIP alone is not applicable for T2DM treatment; however, GLP-1-GIP co-agonists have been recently developed, which present antidiabetic and weight-reducing actions [134].

The current body of evidence suggests that the loss of the incretin effect is secondary to the development of T2DM, and it is not the primary cause of the disturbances in glucose homeostasis [9,12]. The impairment in the incretin effect in diabetic subjects is most likely due to defective pancreas islet response to GIP and GLP-1. The loss of this major mechanism regulating insulin secretion may contribute to the further deterioration of glycemic control and result in glucose toxicity, reduction in β-cell mass, function and expression of GIPR [8].

### 4.2. Incretin Hormones and NAFLD

Non-alcoholic fatty liver disease (NAFLD) is characterized by an excessive hepatic fat accumulation (presence of steatosis in >5% of hepatocytes according to histological analysis) [135]. This condition is closely associated with insulin resistance, which plays a role in both, the pathogenesis of NAFLD and further progression from steatosis to non-alcoholic steatohepatitis (NASH) and fibrosis. NAFLD is present in up to two-thirds of diabetic patients, where the prevalence of this condition is five-fold higher than in nondiabetic patients [136]. Moreover, excessive body weight seems to play a role in the initial process leading to simple steatosis, but also in disease progression [137]. The obesity epidemic was linked with the rising prevalence and severity of NAFLD as this condition has become one of the leading causes of chronic liver disease with the prevalence of 25–30% worldwide, increasing up to 90% in morbidly obese patients [138]. In vitro studies have shown that human hepatocytes express GLP-1R; however, the expression of GLP-1 in the liver tissue is controversial [139]. A study in wild-type and liver-specific *DPP-4* transgenic mice showed that subjects suffering from NAFLD exhibit elevated plasma DPP-4 activity what is associated with diminished glucose-induced active GLP-1 levels [140]. The expression of GIP receptors in the liver is also controversial; however, studies in animal models showed that GIP enhances lipid deposition in the liver, while inhibition of GIP signaling prevents this mechanism [74]. *GIPR* knockout mice had lower triglyceride content and hepatic steatosis on HFD [141], while the study in humans showed that GIP response to saturated fatty acid ingestion is prolonged in nondiabetic patients with NASH and is correlated with liver disease [142].

Weight loss and lifestyle modification is an essential element in NAFLD treatment [135], as 10% reduction of body weight in overweight/obese patients results in significant improvements in insulin sensitivity and 44–58% reduction in hepatic triglyceride content [136]. There is a lack of effective pharmacotherapy for NAFLD treatment; however, anti-obesity medications and agents modulating insulin resistance, including GLP-1, are used for the management of NAFLD [136,138]. The role of incretin mimetics in NAFLD treatment is described in more detail in the further section of this paper.

### 4.3. Incretin Hormones and Cardiovascular Disease

Obesity is associated with over 200 complications, including a significant risk of developing cardiovascular disease, which is associated with excessive visceral fat accumulation and insulin resistance [143]. A large number of studies investigating GLP-1 based intervention have shown beneficial effects of incretin-based therapies on cardiovascular parameters, which are not only associated with improved glycemic control [144]. The expression of GLP-1R has been confirmed in both animal and human models. However, considerable controversy exists over the expression pattern of this receptor in the cardiovascular system, which seems to be species-specific. GLP-1R transcript or protein expression was found in the rat and mouse heart (endocardium, myocardium, microvascular endothelium, and smooth muscle cells in coronary and mesenteric arteries), as well as the human heart (endothelium, smooth muscle cells, coronary arteries, unspecified locations). Discrepancies between results regarding GLP-1R expression may result from different study methodologies, and the specificity of the antibodies used [144,145]. GLP-1 has shown several beneficial effects on the cardiovascular system through different mechanisms. Those include a reduction in blood pressure in several ways: by improving endothelial function, increasing urine excretion and natriuresis, reducing body weight (induced by GLP-1R agonist treatment), activating neural pathways leading to decreased sympathetic nervous system activity, vasodilation due to increased insulin production, and direct vasodilatory action through GLP-1R stimulation in blood vessels [144]. Both native GLP-1 and GLP-1RAs have been shown to reduce cardiac and vascular inflammation [146]. Moreover, preclinical data demonstrated that GLP-1RA might decrease platelet aggregation and increase plaque stability [147,148]. Sternkopf et al. presented that native GLP-1(7–36) can reduce whole blood thrombus formation ex vivo [149]. Further research under physiological conditions is needed to establish whether GLP-1RA exert beneficial effects on parameters of coagulation. Other direct and indirect actions of GLP-1 in the cardiovascular system include increased glucose uptake in the heart, improved left vertical function and beneficial effects on ischemia-reperfusion injury [146]. In most of the cases, the results were obtained after achieving supra-physiological doses of GLP-1, whereas the physiological role of GLP-1 in the cardiovascular system is not known [8]. Most of the studies investigating the role of the incretin system in cardiovascular health-focused mainly on GLP-1; however, studies investigating the role of GIP are emerging. A recent prospective study in two cohorts, including 8044 subjects, showed that higher GIP concentration was associated with risk of higher total mortality (hazard ratio (H): 1.22; 95% CI: 1.11–1.35) and death from CVD (H: 1.3; 95% CI: 1.11–1.52) within 5–9 years of follow-up, whereas GLP-1 levels were not associated with the excess risk [150]. The same team investigated associations between fasting and post-challenge GIP and GLP-1 concentrations and subclinical atherosclerosis (defined by mean intima-media thickness in the common carotid artery and maximal intima-media thickness in the carotid bifurcation) in one of the cohorts. The analysis showed that physiologically elevated levels of fasting GIP are associated with increased mean intima-media thickness in the common carotid artery, whereas GLP-1 concentration was associated with decreased maximal intima-media thickness in the carotid bifurcation. Associations remained significant when diabetic subjects were excluded from the analysis [151]. Results from recent preclinical and clinical studies suggest that GIP may exhibit a blood-pressure-lowering effect and control vasodilation via secretion of nitric oxide. Moreover, GIP influence circulating lipids through its’ effect on adipose tissue uptake and metabolism of lipids and regulate vascular leukocyte adhesion and inflammation via expression and secretion of endothelin 1 [152,153]. Those results suggest that GIP or GIPR signaling may be associated with cardiovascular health. However, further studies are needed to investigate this relationship.

## 5. Genetic Variants of Incretin System in Obesity

*GIPR* and *GLP1R* are genes that encode G protein-coupled receptors for GIP and GLP-1 [154,155]. Genome-wide association studies (GWAS) linked *GIPR* and *GLP1R* variants with BMI and obesity [156,157,158]. The *GLP1R* single nucleotide polymorphism (SNP) rs6923761 (G>A/C) was found to be associated with weight and both anthropometric and several metabolic parameters. In the study of 645 obese nondiabetic Caucasian females, the GG genotype of rs6923761 (wild-type) was associated with higher BMI, body weight, fat mass, waist circumference, triglyceride levels, IL-6, resistin, and leptin levels [159]. Similarly, a study of 1122 obese subjects with (47.4%) and without metabolic syndrome (52.6%) showed that the GG genotype was associated with BMI, weight, fat mass, waist circumference, and waist to hip ratio (WHR). No association was found between the polymorphism and metabolic syndrome or its components [160]. On the contrary, the study of 175 morbidly obese subjects did not find the association between *GLP1R* rs6923761 variant and anthropometric measures; however, the GG genotype of this polymorphism was associated with higher levels of glucose, triglycerides, insulin and HOMA [161]. Another study showed a lack of the association between rs6923761 polymorphism and weight loss or biochemical parameters after two hypocaloric diets—low-carbohydrate vs. low-fat diet. However, obese subjects with the GG genotype had higher BMI, weight and fat mass than A-allele carriers [162]. Other studies by de Luis et al. showed that carriers of mutant allele A of rs6923761 had better anthropometric parameters and lower HOMA, insulin and triglyceride levels, although the lack of association between this polymorphism and weight loss after different hypocaloric diets was shown [163,164,165]. In a group of 137 morbidly obese patients who had biliopancreatic diversion, GG genotype carriers of the rs6923761 showed higher weight loss after bariatric surgery at 12 and 18 months [166]. In addition, the rs2268641 (C>T) SNP of *GLP1R* was associated with obesity in European Americans [158], and *GIPR* SNP rs2334255 (G>T) was identified as a novel common variant associated with both obesity and T2DM risk [167]. An analysis of the coding variants associated with BMI, which included data from 125 studies (718,734 individuals), found two single-nucleotide variants of *GIPR* that contribute independently to lower body weight. Subjects who were carriers of the rs139215588 (G>A) variant weighed 1.92 kg less than noncarriers, whereas carriers of rs143430880 (A>G) variant weighed 1.99 kg less than noncarriers [168]. A study of Hen Chinese found that two SNPs rs11671664 (G>A) and rs2287019 (C>T) of *GIPR* are associated with fat distribution and glucose-related traits [169], and rs11671664 (G>A) was found to be associated with BMI at baseline and body adiposity index (BAI) at baseline and follow-up in the EpiDREAM cohort [170]. In the Look AHEAD Trial, SNP rs11672660 (C>T) of *GIPR* was associated with fewer eating occasions per day [171]. In the POUNDS LOST SNP rs2287019 (C>T) of GIPR was associated with different weight loss diet outcomes—the T allele carriers had greater improvement of glucose homeostasis in individuals who choose a low-fat, high-carbohydrate, and high-fiber diet [172]. The mechanisms explaining how *GIPR* single nucleotide variants influence obesity-related traits remain uncertain; however, it is probable that *GIPR* genetic variants have an impact on the ability of the receptor to stimulate adipogenesis and fat uptake or influence appetite [173].

Moreover, various studies examined the association of *GLP1R* variants with the pharmacological properties of the incretin-based therapies. A pilot pharmacogenetics study examined if allelic rs6923761 (G>A/C) SNP in *GLP1R* modifies the influence of liraglutide and exenatide on gastric emptying. The results showed that minor allele A carriers who were treated with either liraglutide or exenatide had a greater delay in gastric emptying T ½ relative to baseline when compared to GG genotype [174]. In the study investigating weight lowering potential of liraglutide in obese women with polycystic ovary syndrome (PCOS), carriers of GG genotype of rs6923761 had weaker treatment response than non-wild type allele carriers. Moreover, carriers of wild-type CC genotype of rs10305420 (C>T) had a better treatment response than T allele carriers [175]. These results suggest that *GLP1R* and *GIPR* polymorphisms are not only associated with obesity risk but may also influence the effectiveness of incretin-based obesity treatment. Future studies should investigate how genetic variability in *GLP1R* and *GIP* influence the response to incretin-based therapies to explore and support the general objective of personalized medicine.

## 6. Genetic Variants of Incretin System in Obesity-Related Diseases

### 6.1. Genetic Variants of Incretin System in T2DM

Several risk genes for T2DM and related traits have been identified; however, common variants with a relatively small effect size explain only 10–15% of T2DM heritability [176]. Those include variants in *GIPR* and *GLP1R*, which have been shown to be associated with T2DM susceptibility and other traits associated with glucose metabolism. Data from selected studies regarding the variants in *GIPR* and *GLP1R* and their association with T2DM risk and glucose metabolism are shown in Table 1.

Interestingly, polymorphisms of *GIPR* and *GLP1R* genes may interact with macronutrient intake, influencing the glucose homeostasis and the risk of T2DM. A study in the Japanese population showed that *GLP1R* polymorphism rs3765467 (G>A,C,T) was associated with insulin secretion in a nutrient consumption-dependent manner. In this study, men with the GG genotype had decreased insulin secretion in the highest tertiles of energy, protein and carbohydrate consumption [177]. Allele G of rs3765467 variant was also listed as a risk allele for T2DM susceptibility in the Asian population [178].

Similar associations were found for *GIPR* variants. *GIPR* SNP rs2287019 (C>T) was genotyped in 737 overweight adults from the POUNDS LOST trial. Participants were randomly assigned to diets that varied in macronutrient content. In this population, the T allele of rs2287019 was associated with greater improvement of glucose homeostasis in individuals who were assigned to a low-fat diet (20% of energy from fat). There was no significant genotype effect on changes in fasting glucose, insulin and HOMA-IR in the HFD group [172]. Another *GIPR* polymorphism, rs10423928 (T>A), was also shown to interact with diet. In Malmo Diet and Cancer cohort, AA genotype was associated with lower T2DM risk when participants consumed a high-fat and low-carbohydrate diet. In contrast, participants with the TT genotype were at lower T2DM risk when consumed a high-carbohydrate diet [179]. This result was not replicated in the EPIC-InterAct cohort [180].

Genetic heterogeneity in *GIPR* and *GLP1R* may play a role not only in modifying T2DM risk but also in interindividual variability in therapeutic response to diabetic medication. Several *GIPR* variants have been characterized as potentially modulating DPP-4 inhibitor activity; however, this association has not been confirmed by clinical studies. *GLP-1R* is an important drug target for the treatment of T2DM, and several genetic variants in this gene were associated with a response to the glucose-lowering effect of DPP-4 inhibitors, the efficacy of liraglutide and β cell response to GLP-1 administration [181]. Discovery and understanding of pharmacogenetic associations may allow for an individualized approach to T2DM therapy and better health outcomes for patients in the future.

### 6.2. Genetic Variants of Incretin System in NAFLD and CVD

As described in the previous section of this paper, research has linked incretins with cardiovascular health. Evidence that *GIPR* and *GLP1R* polymorphisms may have an influence on cardiovascular biology is also emerging. To date, association with *GLP1R* and CVD has been studied more extensively. An analysis of 17,065 Koreans showed that effect allele A of *GLP1R* variant rs3765467 was associated with decreased risk of CVD [189]. An analysis of 61,846 individuals with coronary heart disease and 163,728 controls of European ancestry showed that SNP rs10305492 (G>A) is not only associated with lower fasting glucose level and lower risk of T2DM, but also a lower risk of coronary heart disease [192]. On the other hand, the Allele A of SNP rs10305445 (C>T) was associated with increased frequency of cardiovascular events within five years of follow-up in hemodialysis patients with chronic kidney disease [193]. In T2 diabetic patients from a Han Chinese population, GG genotype of *GLP1R* rs4714210 (A>G) polymorphism was associated with a lower risk of developing coronary artery disease [194]. One of the variants of *GIPR* was also associated with cardiovascular health. Nitz et al. analysis of subjects from the EPIC-Potsdam study showed that heterozygous minor allele C of rs1800437 (G>C) variant is associated with CVD [182].

To date, the association of *GIPR* and *GLP1R* polymorphism with NAFLD has not been studied.

## 7. Incretin Based Therapy for Obesity Treatment

**Figure 4:**
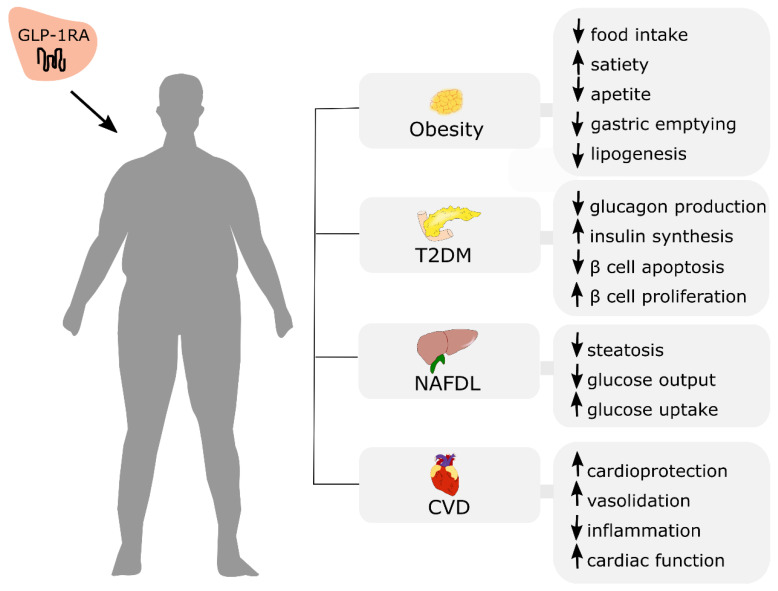
Potential therapeutic effects of GLP-1RA administration in obese patients with co-existing cardiometabolic disorders. T2DM: type 2 diabetes mellitus; NAFLD: non-alcoholic fatty liver disease; CVD: cardiovascular disease. ↑: increase; ↓: decrease.

Incretin-based therapies offer great therapeutic potential, not only for initially established T2DM treatment but also obesity and the management of other noncommunicable diseases, including NAFLD and cardiovascular disease (Figure 4).

GLP-1R agonists have documented effects on reduced food intake and weight loss. A meta-analysis of eight randomized controlled trials investigating the weight-lowering effect of GLP-1 agonists in individuals with overweight or obesity (without diabetes) showed that administration of GLP-1 mimetics (liraglutide or exenatide) resulted in larger weight loss in comparison to the control group. Moreover, GLP-1A reduced BMI, waist circumference, systolic blood pressure (SBP) and triglyceride level [195]. This result was similar to the conclusion of the previous meta-analysis, including 21 trials where treatment of obese patients (with or without diabetes) with GLP1A (liraglutide or exenatide twice daily or once weekly) resulted in an average weight loss of 2.9 kg (95% CI −3.6 to −2.2) [196]. Despite the fact that various GLP-1RAs were shown to promote weight loss [197], currently, only administration of liraglutide 3.0 mg is approved by the Food and Drug Administration (FDA) and European Medicines Agency (EMA) for the treatment of obesity [198,199]. The satiety and clinical adiposity liraglutide evidence (SCALE), which was a clinical development program, investigated the safety and efficacy of liraglutide 3 mg in individuals with and without diabetes. Depending on the trial, participants experienced a dose-dependent weight loss, with similar outcomes between races. Weight loss varied from 6.3% in the Asian and African-American population to 7.7% in white, where individuals from placebo groups had a mean weight loss of 0.49–2.5% [200]. The most common side effects associated with liraglutide administration include nausea (25.0%), vomiting (12.2%), diarrhea (11.6%), constipation (11.0%), and dyspepsia (6.4%) [201]. Other anti-obesity drugs that are emerging include exenatide and semaglutide. Exenatide is a short-acting GLP-1 analog that has completed a phase 3 randomized controlled trial for obese individuals without diabetes. The results of the studies are still equivocal; therefore, further research is needed to establish if exenatide can be used for obesity treatment. Semaglutide is another GLP-1 analog (long-acting) that promotes weight loss. Various studies showed that administration of semaglutide results in significant weight loss vs. placebo [201]. Further clinical trials will investigate whether treatment with semaglutide will reduce the risk of cardiovascular events in overweight and obese patients with co-existing CVD.

There is a number of other potential incretin-based therapies that have reached phase 2 clinical trials. One of them includes treatment with efpeglenatide—a long-lasting GLP-1R agonist. In a randomized, double-blind placebo trial, participants with BMI ≥ 30 kg/m^2^ or ≥27 kg/m^2^ with comorbidity were randomized to groups receiving different efpeglenatide doses or placebo in combination with a hypocaloric diet. Over 20 weeks, all doses of efpeglenatide significantly reduced body weight from baseline versus placebo. The highest placebo-adjusted weight loss was −7.2 kg for a group receiving 6 mg of efpeglenatide once weekly. Gastrointestinal effects were the most common side effects [202]. Another medication that can be a potential anti-obesity drug is MEDI0382—a GLP-1 and glucagon receptor dual agonist. In a randomized, double-blind placebo trial recruiting overweight and obese diabetic patients, administration of MEDI0382 resulted in a significant weight loss of −3.84 kg (vs. −1.7 kg in the placebo group) after 41 days [203]. Current data suggest that in humans, glucagon-GLP-1 agonists are as effective as GLP-1 agonists but not superior [204]. Another promising therapy for weight loss is the combination of GIPR and GLP-1R agonists. One of the molecules that represent this group is tirzepatide (LY329817), which results in weight loss and reduced glucose level and food intake [204]. SURPASS clinical trials aimed to assess the efficacy and safety of tirzepatide treatment to improve glycemic control in people with T2DM. To date, tirzepatide has demonstrated dose-dependent weight loss reduction (up to −11.3 kg with 15 mg dose) in diabetic patients. Further studies, including SURPASS-2 and SURMOUNT-1, will investigate if GIP-GLP-1 co-agonists are more effective in reducing weight and HbA1c than GLP-1 agonists alone in diabetic and nondiabetic populations [205]. Other therapies in phase 1 clinical trials include combination therapies, where GLP-1/glucagon/GIP receptor triple agonists were developed to enhance GLP-1 actions [201]. Administration of GLP-1/glucagon/GIP triple-agonist (HM15211) in diet-induced obese mice resulted in significant weight loss after four weeks relative to liraglutide dose equivalent to 3 mg in humans [206].

## 8. Incretin Based Therapy for Obesity-Related Metabolic Disorders

### 8.1. Incretin-Based Therapy for Type 2 Diabetes Treatment

#### 8.1.1. Mechanisms of Action

A number of non-insulin based oral therapies is used for T2DM treatment, including incretin-based therapies. They include GLP-1 analogs and receptor agonists, and DPP-4 inhibitors. GLP-1 analogs and receptor agonist administration are associated with increased insulin secretion and inhibition of glucagon release, resulting in a blood glucose-lowering effect [207]. As described in the previous section of this paper, GLP-1 and its agonists also inhibit gastric emptying. This action is associated not only with the satiating effect but also a reduction in glycemia. Several studies confirmed that administration of GLP-1 and GLP-1RAs inhibit gastric emptying in T2 diabetic patients, and this effect may contribute to the blood-glucose-lowering properties of GLP-1, together with the stimulation of insulin and the inhibition of glucagon secretion [208,209,210,211]. Slower gastric emptying, which impacts glucose absorption and, therefore, postprandial glycemia, was also observed in healthy individuals [210,212,213]. Due to the presence of an alanine residue at the N terminus, GLP-1 is metabolized by DPP-4 after 1–2 min. New analogs of GLP-1 were designed by substitution of the alanine group with other amino acids, so they are resistant to DPP-4 degradation. They are recommended as an add-on therapy for patients who did not benefit sufficiently from metformin, do not tolerate or cannot be considered for the treatment with this medication [214]. Due to specific mechanisms of action, incretin mimetics are characterized by a low risk of hypoglycemia; however, one of the disadvantages of those peptide-based therapies is that they need to be administrated by subcutaneous injection, just like insulin, to avoid degradation by gastrointestinal enzymes [215].

DPP-4 is an enzyme that is responsible for the degradation of GLP-1 and other biologically important peptides. DPP-4 inhibitors restrain ≥ 90% of DPP-4 activity, therefore increasing the half-life of physiological GLP-1 [215]. DPP-4 inhibitors are administrated orally and have a similar glucose-lowering effect, with HbA1c lowering efficacy established to be around 0.5–1.0% (when used for a long period) [216]. This class of medication demonstrates good safety and tolerability, where the most frequent side effects include nasopharyngitis and skin lesions [217]. There has been concern that GLP-1RAs and DPP-4 inhibitors administration may be associated with a higher risk of pancreatitis and pancreatic cancer. Recent research did not explicitly establish this association for neither GLP-1 mimetics [218] nor for DPP-4 inhibitors [219,220]. Therefore, further studies are needed to establish the risk of pancreatitis correlated with those therapies.

Both GLP-1RA and DPP-4 inhibitors reduce HbA1c; however, GLP-1RA are more effective in lowering glycemia, and their use is additionally associated with weight loss. Long-acting GLP-1RAs lower HbA1c more effectively than short-acting agents, but the weight loss effect is similar [221]. The Consensus of the American Diabetes Association (ADA) and European Association for the Study of Diabetes (EASD) recommends GLP-1RA and DPP-4 inhibitors as therapeutics for T2DM treatment. They can be considered as an alternative to or in combination with metformin when HbA1c targets are not reached. Moreover, if weight gain needs to be minimized or weight loss is recommended, GLP-1AR with good efficacy for weight loss are recommended (semaglutide > liraglutide > dulaglutide > exenatide > lixisenatide). Patients with CVD or at high cardiovascular risk should be considered for GLP-1RA treatment with proven CVD benefit [222].

The research did not show obvious therapeutic potential for GIP in T2DM, as it seems to have a minor effect on insulin secretion in those patients; it increases glucagon secretion and does not show glucose-lowering properties even at supra-physiological doses [8]. Although the clinical investigation of GIP analogs has been overshadowed by GLP-1 analogs, the recent research showed the potential benefits of combining GIP with GLP-1. Currently, chimeric peptides combining GIP with GLP-1 and GIP, GLP-1 and glucagon are under investigation as a potential treatment of obese patients with T2DM [223].

#### 8.1.2. Incretin-Based Therapies for T2DM

Exenatide was the first GLP-1RAs that was registered for T2DM treatment, which was approved by the FDA in 2005. DPP-4 inhibitors were introduced one year later when sitagliptin was first registered [217,224]. To date, seven GLP-1 mimetics and five DPP-4 inhibitors were approved for T2DM treatment with some differences of approved drugs across different countries [18]. Their names and characteristics are presented in Table 2.

Currently, other incretin-based therapies are under investigation. Those include studies of a long-acting GLP-1RA efpeglenatide and dual GIP/GLP-1 agonist tirzepatide. The latter was developed by Eli Lilly and is a synthetic linear peptide containing 39 amino acids, which can be administrated subcutaneously once weekly. Several SURPASS trials investigated the efficacy and safety of tirzepatide for T2DM treatment. To date, phase 1 and 2 trials demonstrated a dose-dependent reduction in HbA1c up to 2.4%. Further phase 3 trials will investigate the long-term efficacy, safety and cardiovascular outcomes of this medication [205]. Novel therapies based on peptide agonists combining the activity of GLP-1, GIP and glucagon are also under investigation. To date, several animal model studies showed that those multi-target treatments are more effective than single-target therapy [225,226,227,228]. Further clinical studies will allow investigating the safety and beneficial effects of those drugs in humans.

### 8.2. Incretin-Based Therapy for NAFLD Treatment

#### 8.2.1. Mechanisms of Action

Several animal and human studies showed the therapeutic effects of incretin-based therapies in slowing the progression of NAFLD [231]. A study in rats fed HFD showed that administration of GLP-1RA—exenatide was associated with improved fatty acid β-oxidation and insulin sensitivity in the liver. This effect resulted from an increase in PKA activity, Akt and AMPK phosphorylation and contributed to PKA-dependent increase of peroxisome proliferator-activated receptor α activity [232]. Another mechanism in which GLP-1 could improve hepatic lipid metabolism is the regulation of nicotinamide phosphoribosyltransferase, sirtuin 1 and AMPK signaling [231]. Moreover, incretin mimetics have been shown to directly reduce hepatocyte steatosis by modulating elements of the insulin signaling pathway [233]. The treatment with liraglutide in animal models has been recently associated with the improvement in hepatic steatosis by downregulation of the expression of inflammatory signaling mediators [234] and by regulating the local renin–angiotensin system [235].

#### 8.2.2. Incretin-Based Therapies for NAFLD

The efficacy and safety of GLP-1RAs have been assessed by several systematic reviews. In one of the most recent reports, Sofogianni et al. investigated the role of exenatide, lixisenatide, dulaglutide, liraglutide, and semaglutide in the management of NAFLD. Among all of the different agents, liraglutide and exenatide have been studied the most extensively. Those two GLP-1RAs show an improvement in the liver function tests and histology, exert hepatoprotective and glucose-lowering actions, and their administration is associated with decreased hepatic and visceral fat accumulation. There is limited data available on the use of lixisenatide and dulaglutide in NAFLD patients. However, the latter present similar therapeutic improvements to liraglutide. Semaglutide seems to be the most promising GLP-1RA for NAFLD treatment as its use is associated with the prevention of cardiovascular events. Moreover, when compared to dulaglutide, it shows better glucose control and weight reduction in diabetic patients. The authors of the review concluded that GLP-1RA might be a promising treatment for NAFLD patients; however, further studies are needed to confirm this correlation [236]. Teshome et al. also verified the efficacy of GLP-1RA in NAFLD treatment; however, only studies using liraglutide and exenatide were included in this review paper. The authors concluded that compared with baseline, body weight, alanine aminotransferase (ALT), aspartate aminotransferase (AST), and gamma-glutamyltransferase (GGT) were decreased by 5.5%, 59.5%, 52.8%, and 44.8%, respectively, due to GLP-1RA treatment [237]. Similar results were obtained by Kumar et al. in the meta-analysis, including 946 patients with NAFLD. GLP-1RA administration was associated with a reduction in BMI, steatosis, and ALT and GGT levels. There were no significant changes in AST levels [238]. There are limited data on the effectiveness of DPP-4 inhibitors in NAFLD treatment. A recent meta-analysis by Kumar et al. showed that their use is associated with a significant reduction in ALT; however, no significant changes in AST, GGT, fibrosis, steatosis, and BMI were observed [238]. Other most recent systematic reviews, including studies where NAFLD patients were treated with DPP-4 inhibitors (sitagliptin or vildagliptin), also concluded that those agents exert no beneficial effects on liver transaminase and steatosis in this group [239,240,241]. A recent study demonstrated that dual GIP and GLP-1 agonist tirzepatide significantly improves NASH-related biomarkers in the T2DM population. Analysis of 316 diabetic patients showed a greater decrease in ALT in groups treated with tirzepatide 10 and 15 mg than with dulaglutide. Further research should evaluate the efficacy of tirzepatide in patients with NASH [242].

Current guidelines do not recommend pharmacological therapy for patients in the initial stage of NAFLD, where pharmacotherapy should be reserved for more advanced disease, e.g., progressive NASH. Guidelines acknowledge that any medications prescribed for NAFLD should be considered as an off-label treatment, where GLP-1 analogs have been classified as potentially useful [243]. Further studies are needed to prove the efficacy and safety of incretin-based therapies for NAFLD treatment.

### 8.3. Incretin-Based Therapy for CVD Treatment

Since 2008, FDA requires all new diabetes therapies to demonstrate cardiovascular safety; therefore, cardiovascular outcomes trials have been carried out to establish the cardiovascular safety of incretin-based therapies [244]. To date, four GLP-1RAs have been proven to reduce the risk of major adverse cardiovascular events (MACE) in patients at high cardiovascular risk or with established cardiovascular disease. They include liraglutide, semaglutide, albiglutide and dulaglutide, whereas lixisenatide and extended-release exenatide have a neutral effect on the cardiovascular system [245,246]. LEADER trial investigated cardiovascular outcomes in 9340 diabetic patients treated with liraglutide or placebo, with a median follow-up of 3.8 years. Liraglutide treatment was associated with reduced both all-cause mortality (H: 0.85, 95% CI: 0.74–0.97) and the 3-point MACE (13.0% vs. 14.9% in the placebo group, respectively). The differences between the liraglutide group and the placebo group in the rates of nonfatal myocardial infarction, nonfatal stroke, and hospitalization for heart failure were non-significant [247]. A post hoc analysis from SCALE randomized trials included 5908 overweight or obese participants who did not show a decrease in cardiovascular risk. In this analysis, treatment with liraglutide 3.0 mg was associated with a mean increase in pulse, a decrease in SBP and no change in cardiovascular risk [248]. SUSTAIN-6 trial investigated the cardiovascular safety of injectable semaglutide in 3297 diabetic patients with a median follow-up of 2.1 years. The results showed that semaglutide reduced the rate of 3-point MACE by 26%, which was driven by a significant reduction in nonfatal stroke and a non-significant decrease in nonfatal myocardial infarction. Rates of death from cardiovascular causes were similar in placebo and semaglutide groups [249]. PIONEER 6 was the second trial for semaglutide; however, cardiovascular safety was established for the oral formulation of this drug. A total of 3138 subjects with T2DM were included in the study and assigned either to placebo or semaglutide group. The results were suggestive of a cardiovascular benefit of semaglutide; however, superiority was not achieved [250]. A post hoc analysis of both, SUSTAIN-6 and PIONEER 6 trials data combined showed that semaglutide was superior to placebo for the reduction in the incidence of MACE [251]; however, only injectable semaglutide was approved in the United States for the new label indication [246]. HARMONY trial included 9463 diabetic patients treated with either albiglutide or placebo with a median follow-up of 1.6 years. The results showed that albiglutide reduced the risk of the primary outcome—death from a cardiovascular cause, nonfatal myocardial infarction, or nonfatal stroke by 22%, in comparison to placebo [252]. REWIND trial included 9901 diabetic subjects treated with either dulaglutide or placebo, with a median follow-up of 5.4 years. The results showed that treatment with dulaglutide was associated with reduced the relative risk of MACE-3 by 12% (H: 0.88, 95% CI: 0.79–0.99) [253]. The evaluation of lixisenatide in acute coronary syndrome (ELIXA) and the Exenatide study of cardiovascular event lowering (EXSCEL) trials did not show the superiority of those two agents over placebo; however, they met the goal for cardiovascular safety [254,255]. Interestingly, a recent meta-analysis of four of the mentioned trials (ELIXA, LEADER, SUSTAIN-6 and EXSCEL) showed that all GLP-1RA significantly reduced all-cause mortality and cardiovascular mortality in comparison to placebo. When only long-acting GLP-1RA were analyzed (liraglutide, semaglutide and extended-release exenatide), a significant reduction in major adverse cardiac events and nonfatal strokes was also seen. The authors suggested that the discrepancies in the trial outcomes may be due to differences in pharmacokinetic and pharmacodynamic properties, as well as disparities in study designs [256]. Potential underlying mechanisms associated with the reduction of cardiometabolic risk by GLP-1RA remain largely unknown; however, multifactorial effects including improved glucose control, lower blood pressure, bodyweight reduction, and possibly improvement in other parameters related to cardiovascular risk have been suggested [257]. While four GLP-1RA exhibit cardioprotective properties, only liraglutide 3 mg has been registered for obesity treatment. GLP-1RA currently approved for the treatment of T2DM may offer safe and effective treatment of obese patients with or at high risk of CVD; however, further studies are needed.

DPP-4 inhibitors seem to have a neutral impact on cardiovascular outcomes [258,259]; however, there is evidence for a higher risk of hospitalization from heart failure for saxagliptin in the overall population [260,261]. As described in the previous section of this paper, GIP may also play a role in cardiovascular physiology and pathophysiology. It is still unclear whether GIPR agonisms or antagonisms may have a therapeutic effect in cardiovascular disease [152]; however, recent phase 2 clinical data on the effect and safety of the GIPR/GLP-1R dual-agonist tirzepatide in diabetic patients showed significant weight loss, improved glycemic control, and decrease of total cholesterol and triglyceride levels [262]. Subsequent analysis of the samples from the same study showed the additional effect of tirzepatide on the levels of apolipoproteins and lipoprotein particle subclasses, suggesting an improvement in atherogenic lipoprotein profile [263]. Further studies investigating GIPR agonisms and antagonisms and their effect on cardiovascular health are needed.

## 9. Conclusions and Future Clinical Perspectives

Incretin-based therapies offer great therapeutic potential for obesity treatment and the management of its comorbidities, including T2DM, NAFLD and cardiovascular disease. The prevalence of those disorders is increasing, and obese patients often suffer from all of the above-mentioned conditions. GLP-1RA have been shown to be very effective in long-term weight loss and HbA1c reduction, while recent research indicates that obese and diabetic patients with other comorbidities may also benefit from their cardioprotective and hepatic steatosis reducing properties. The therapeutic potential of GIP is not obvious, as even supra-physiological doses of this incretin are not associated with glucose-lowering properties. Further research is needed to establish GIP applicability in incretin-based therapies. Currently, new drug classes of agents are being developed. They include dual incretin receptor agonists and GLP-1/glucagon/GIP receptor triple agonists, which may offer better blood glucose and weight loss control than currently available single-agonist treatments. Despite promising results, inconclusive findings still exist, and some mechanisms of action of incretin hormones on different systems and organs remain unclear. Future research should focus on understanding the interactions between gut hormones and the nervous system, endocrine cells, genetic background, composition and intake of food to establish how they affect individual response to treatment of obesity and related cardiometabolic disorders.

## Figures and Tables

**Table 1:** Selected variants in *GIP* and *GLP1R* and their association with T2DM risk and glucose metabolism.

Gene	SNP	Alleles	Consequence	Associated traits	Population	Author
*GIPR*	rs1800437	G/C	Missense variant	No association with T2DM	EPIC-Potsdam, Germany	Nitz et al. [182]
				No association with T2DM	Egyptian	Shalaby et al. [183]
	rs2302382	C/A	Splice region variant	Allele A was associated with T2DM risk	Egyptian	Shalaby et al. [183]
	rs10423928	T/A	Intron variant	Allele A was associated with decreased insulin level after OGTT and increased risk of T2DM	GWAS meta-analysis	Saxena et al. [184]
				Allele A was associated with an increased risk of T2DM	Chinese	Hu et al. [185]
				Allele A was associated with higher fasting proinsulin levels and a lower insulinogenic index	White European and US cohort	Ingelsson et al. [186]
	rs8108269	T/G	Intergenic variant	No association with T2DM	Japanese	Matsuba et al. [187]
				Allele G identified as a risk allele for T2DM susceptibility	European, GWAS	Morris et al. [188]
	rs2334255	G/T	3′ UTR variant	Polymorphism associated with T2DM and obesity	various, GWAS	Zhang et al. [167]
*GLP1R*	rs3765467	G/A/C/T	Missense variant	Effect allele A was associated with decreased risk of T2DM, decreased glucose level and HbA1c in control subjects without diabetes	East Asian	Kwak et al. [189]
				Allele G identified as a risk allele for T2DM susceptibility	Japanese	Suzuki et al. [178]
	rs10305492	G/A	Missense variant	Allele A was associated with lower fasting glucose level and a lower risk of T2DM	-	Scott et al. [190]
				Allele A was associated with lower fasting glucose and T2DM risk	Various, GWAS	Wessel et al. [191]
	rs367543060	C/T	Missense variant	No association with T2DM	Egyptian	Shalaby et al. [183]
	rs6923761	G/A/C	Missense variant	No association with fasting glucose	Caucasian females (diabetic)	De Luis et al. [159]
				Allele A was associated with higher insulin and HOMA levels	Morbidly obese patients	De Luis et al. [161]

T2DM: type 2 diabetes mellitus; OGTT: oral glucose tolerance test; GWAS: genome-wide association study; HbA1c: glycated hemoglobin; HOMA: homeostatic model assessment.

**Table 2:** Incretin-based therapies approved for T2DM [18,221,229,230]. Modification.

Drug	Homology to Native GLP-1/DPP-4 Inhibition	T_1/2_	Dosage	Reduction in HbA1c (%)	Reduction in Body Weight (kg)
GLP-1RA					
Exenatide extended-release	53%	96 h	2 mg once weekly	1.3–1.9	2–3.7
Exenatide immediate release	53%	2.4 h	5–10 μg twice daily	0.8–1.2	1–3
Liraglutide	97%	~13 h	1.2–1.8 mg once daily	0.8–1.5	2–3
Dulaglutide	90%	~1 week	0.75–1.5 mg once weekly	0.78–1.5	0.8–2.5
Semaglutide	94%	4.5–4.7 days	0.5–1 mg once weekly	1.2–18	3.5–6.5
Lixisenatide	50%	~3 h	20 μg once daily	1.3–2.7	1.3–2.7
Albiglutide	97%	5 days	30–50 mg once weekly	0.7–1	0.8–1.1
DPP-4 inhibitors					
Sitagliptin	Max: ~97%, 80% 24 h post dose	8–24 h	100 mg once daily	0.4–1.3	-
Saxagliptin	Max: ~80%; 70% 24 h post dose	2–4 h	5 mg once daily	0.5–1.7	-
Linagliptin	Max~80%; 70% 24 h post dose	10–40 h	5 mg once daily	0.2–1.1	-
Alogliptin	Max: ~90%; 75% 24 h post dose	12–21 h	25 mg once daily	0.5–0.7	-
Vildagliptin	Max: ~95%; 80% 24 h post dose	1.5–4.5 h	50 mg twice daily	0.2–1	-

DPP-4: dipeptidyl peptidase-4; T_1/2_: half-life; HbA1c: glycated hemoglobin; GLP-1RA: glucagon-like peptide-1 receptor agonist.

## Data Availability

No new data were created or analyzed in this study. Data sharing is not applicable to this article.
